# Morphological aspects of the skin in felines with leishmaniasis: a histopathological study

**DOI:** 10.1590/S1984-29612026013

**Published:** 2026-06-15

**Authors:** Ianete Lima Batista, Richard Átila de Sousa, Joílson Ferreira Batista, Márcia dos Santos Rizzo, Luciana Pereira Machado, Carlos Henrique Nery Costa, Ivete Lopes de Mendonça

**Affiliations:** 1 Universidade Federal do Piauí – UFPI, Programa de Pós-graduação em Tecnologias a Animas de Interesse Regional, Teresina, PI, Brasil; 2 Universidade Federal do Piauí – UFPI, Departamento de Clínica e Cirurgia Veterinária, Teresina, PI, Brasil; 3 Universidade Federal do Piauí – UFPI, Departamento de Morfologia, Teresina, PI, Brasil; 4 Universidade Federal da Fronteira do Sul – UFFS, Departamento de Medicina Veterinária, Realeza, PR, Brasil; 5 Universidade Federal do Piauí – UFPI, Departamento de Medicina Comunitária, Teresina, PI, Brasil

**Keywords:** Dermatopathology, cat, Leishmania, macrophages, Dermatopatologia, gato, Leishmania, macrófagos

## Abstract

Felines are susceptible to*Leishmania*spp. infection in endemic areas; however, histopathological studies on the organs of infected felines remain scarce. This study evaluated the inflammatory response and cutaneous parasitism in 13 naturally infected felines in Teresina, Piauí, Brazil. After diagnosis, the animals were euthanized, and skin fragments from the face, external ear, periocular region, muzzle, and dorsum were histopathologically analyzed. The cytology of the ear skin revealed amastigote forms of*Leishmania*spp. in 84.62% of the felines, with this region being the most parasitized. The inflammatory infiltrate was mild-to-moderate in 38.5%, with a diffuse distribution in 77% of cases. Mononuclear cells predominated in 92%, while polymorphonuclear cells were rare (8%). Amastigote forms were discrete in 46%, and 46% presented with granulomatous dermatitis. The main epidermal changes were exocytosis (92.5%), acanthosis, and spongiosis (61.5%). These findings highlight the importance of histopathology as a diagnostic tool and contribute to the understanding of the pathogenesis of feline leishmaniasis, aiding in the development of diagnostic and therapeutic research.

## Resumo

Os felinos são suscetíveis à infecção por*Leishmania*spp. em regiões endêmicas; no entanto, estudos histopatológicos sobre os órgãos de felinos infectados ainda são escassos. Este estudo avaliou a resposta inflamatória e o parasitismo cutâneo em 13 felinos naturalmente infectados em Teresina, Piauí, Brasil. Após o diagnóstico, os animais foram eutanasiados, e fragmentos de pele da face, orelha externa, região periocular, focinho e dorso foram analisados histopatologicamente. O exame citológico da pele da orelha revelou formas amastigotas de*Leishmania*spp. em 84,62% dos felinos, sendo essa a região mais parasitada. O infiltrado inflamatório foi classificado como discreto a moderado em 38,5% dos casos, com distribuição difusa observada em 77% das amostras. Células mononucleares predominaram em 92% dos casos, enquanto células polimorfonucleares foram raras (8%). Formas amastigotas foram pouco observadas em 46% das amostras. E 46% apresentaram dermatite granulomatosa. As principais alterações epidérmicas incluíram exocitose (92,5%), acantose e espongiose (61,5%). Esses achados destacam a importância da histopatologia como ferramenta diagnóstica e contribuem para a compreensão da patogênese da leishmaniose felina, fornecendo subsídios para o avanço de pesquisas diagnósticas e terapêuticas.

## Introduction

Feline leishmaniasis (FeL) occurs in 99 countries and is caused by protozoa of the genus*Leishmania*, transmitted by vectors (WHO, 2021). In Brazil, felines are recognized as potential reservoirs of the parasite (Silva et al., 2010; Mendonça et al., 2020), with*Leishmania infantum*being the predominant species in areas endemic to humans and dogs (Mendonça et al., 2017). The skin—composed of keratinocytes, dendritic cells, monocytes/macrophages, and granulocytes—acts as a physical barrier against exogenous agents (Pennisi & Persichetti, 2018).

In cats, the infection is often subclinical. Clinical signs include papules, nodules, ulcers, erythema, and alopecia (Simpson et al., 2011). Cutaneous and mucocutaneous nodules—usually small (≤1 cm), painless, and mildly pruritic—are common in regions such as the head (eyelids, nose, and lips), distal limbs, and anal mucosa (Pennisi et al., 2013). Ulcers may be superficial or deep and occurring in the same areas, with the head, ears, muzzle, and periocular regions being the most affected, possibly because these areas are preferred by sandflies for blood feeding (Migliazzo et al., 2015).

Histopathology plays a crucial role in addressing knowledge gaps regarding clinical and pathological manifestations, improving diagnosis and monitoring, and guiding new therapeutic strategies. Therefore, the aim of the study is to perform histopathological analysis of the skin of felines with leishmanaises.

## Material and Methods

Thirteen mixed-breed felines of both sexes and varying ages, from Teresina, Piauí, Brazil, were used. The project was approved by the Ethics Committee on Animal Use (CEUA/UFPI) under protocols nº 437/2018 and 735/2022 and conducted at the Interdisciplinary Laboratory of Advanced Materials of the Federal University of Piauí. Eleven felines were naturally infected with*Leishmania infantum*, as confirmed in previous studies (Batista et al., 2020), and two were diagnosed with*Leishmania*spp. in this study, as confirmed by parasitological tests of lymph nodes and blood smears. All included animals underwent clinical evaluation.

After euthanasia with 2.5% sodium thiopental (80 mg/kg) and 20% potassium chloride, administered intravenously, the animals were necropsied for histopathological analysis hematoxilin-eosin. Skin samples from the ear, muzzle, face, periocular region, paws, and dorsum were fixed in 10% buffered formalin. Histopathological changes were evaluated for intensity (mild, moderate, or severe) and distribution (absent, focal, multifocal, or diffuse), considering the cellular infiltrates, cell types, presence of amastigote forms, and inflammatory processes. The intensity of the infiltrate was classified as absent (-), mild (+), moderate (++), severe (+++).

## Results

Of the 13 cats evaluated, 84.62% presented clinical signs, while 15.38% were asymptomatic (Figure 1). The most common systemic alterations were popliteal lymphadenomegaly (76.9%), alopecia (53.8%), and weight loss (38.4%). Ocular manifestations were rare, with uveitis being the most common (23.0%). Generalized alopecia and scaling occurred in 57.1%, while 42.9% presented localized alopecia, mainly on the head, ears, periocular region, muzzle, and lips. Nodules on the ear tip were identified in 23.0% of cases (Figure 2).

**Figure 1:**
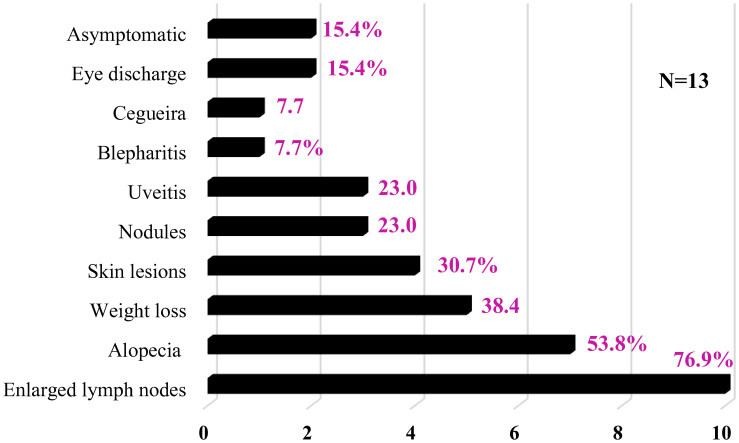
Dermatological and clinical symptoms observed in felines naturally infected with*Leishmania*spp., residing in Teresina, Piauí, Brazil. The clinical signs are not mutually exclusive, and the same animal may present with more than one manifestation.

**Figure 2:**
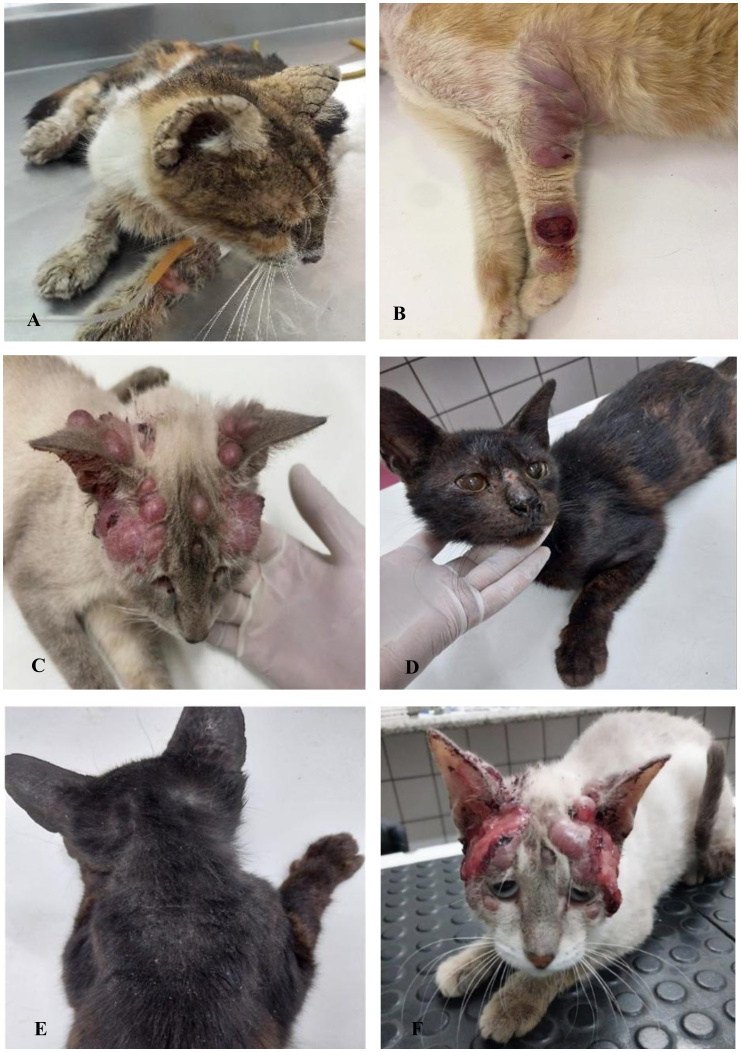
(A) Hyperkeratosis and lignification; (B) Hardened subcutaneous nodules, measuring approximately 1 to 2 cm in diameter, located on the medial aspect of the thoracic limb, involving the distal region of the forearm and the carpal region; (C) Multiple, non-ulcerated, alopecic nodules with scales on the face and ear; (D) Lesion on the snout; (E) Area of ​​alopecia on the back with hair loss and scaling of the skin; (F) Multiple ulcerated alopecic nodules on the face and ears.

Histopathological analysis revealed that all 13 felines exhibited inflammatory infiltration in at least one evaluated skin segment. Segments of the ear, muzzle, dorsum, paw, periocular region, and face, including areas with and without dermal alterations, were examined. The infiltrate intensity ranged from mild to moderate (38.5%), whereas severe infiltration was observed in 23.1% of cases. The highest concentration of inflammatory cells occurred in the ear (30.76%; +++).

Diffuse infiltration was predominant (77%), especially in the ear (30.76%), whereas focal infiltration was observed in 23% of cases. Amastigote forms of*Leishmania*spp. were identified both inside and outside the macrophages, with intensities ranging from mild to severe, depending on the region. The ear showed the highest parasitism (53.84%), followed by the dorsum (46.15%), while the paw had a moderate parasitic load (7.69%). Parasitic forms were not evident on the face in 7.69% of cases, while 46% presented with leishmaniasis-associated granulomatous dermatitis, Table 1.

**Table 1:** Histopathological findings observed in the dermis of felines with leishmaniasis.

**DERMIS – INFLAMMATORY INFILTRATE**											
	**Intensity**			**Distribution**		**Cellular Type**		**Amastigote**			**G.F**
	Dis	Mod	Int	Foc	Dif.	PMN and MN	MN	Dis	Mod	Int	
Number	5/13	5/13	3/13	3/13	10/13	1/13	12/13	6/13	2/13	5/13	6/13
Percentage	38,5%	38,5%	23,1%	23%	77%	8%	92%	46,2%	15,4%	38,5%	46%

Dis = discrete; Mod = moderate; Int = intense; Foc = focal; Dif = diffuse; PMN = polymorphonuclear cells; MN = mononuclear cells, G.F = granuloma formation.

Among epidermal alterations, exocytosis was the most frequent (92.3%). Acanthosis and spongiosis were identified in 61.5% of the samples. Ulcers were detected in 15.4% cases, while parakeratosis was the least common, occurring in only 7.7%, Table 2. A relevant observation was the higher occurrence of exocytosis in muzzle fragments (7.69%), followed by the ear and dorsal regions (6.41%) (*N* = 78). Acanthosis was frequently observed in the muzzles (3.84%) and ears (5.12%). Parakeratosis was rare and identified in the cutaneous tissue of the dorsum of only one animal. Figures 3 and 4 represent the skin lesions of felines with leishmaniasis, presenting both epidermal and dermal alterations.

**Table 2:** Histopathological analysis of the skin of felines naturally infected with*Leishmania*spp.

	**Ulcer**	**Acanthosis**	**Spongiosis**	**Exocytosis**	**Paraqueratosis**
Number	02/13	08/13	08/13	12/13	01/13
Percentage	15.4%	61.5%	61.5%	92.3%	7.7%

**Figure 3:**
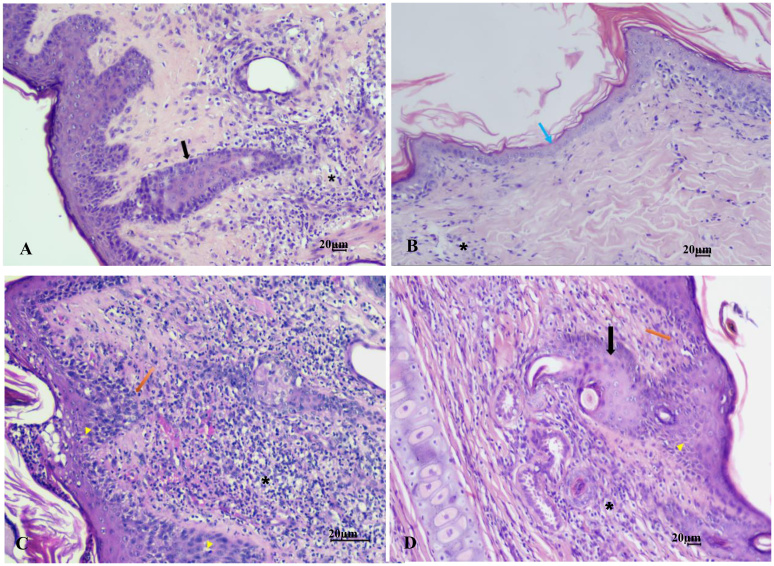
Photomicrographs of skin lesions in felines with cutaneous leishmaniasis, displaying epidermal and dermal alterations (A-D). Staining: H&E. Scale bar: 20 µm. The epidermis shows irregular acanthosis (black arrow), slight thinning of the epidermis (blue arrow), lymphohistiocytic exocytosis (orange arrow), and spongiosis (yellow arrowhead). Dermis with a predominantly diffuse infiltrate of mononuclear cells (*asterisk).

**Figure 4:**
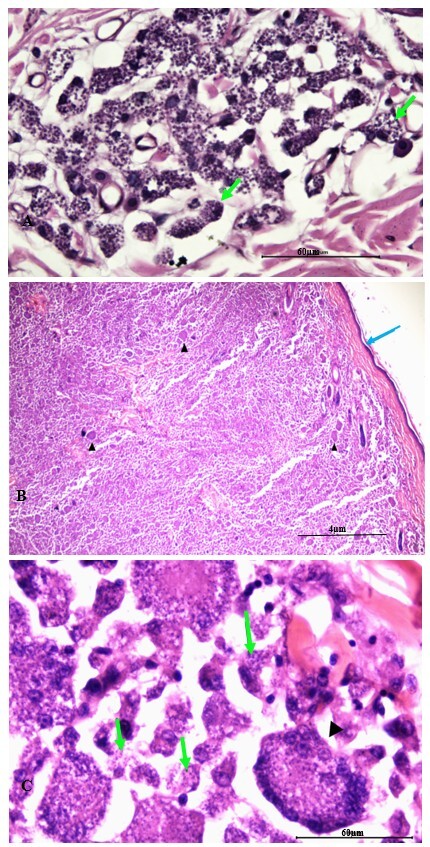
Photomicrographs of skin lesions in felines with cutaneous leishmaniasis. Staining: H&E. Scale bar: 4 µm and 60 µm. (A) Numerous intracellular amastigotes of*Leishmania*within macrophages (green arrows) present in the inflammatory infiltrate in the dermis; (B) Nodular to diffuse dermatitis with granuloma formation in a papular skin lesion, showing numerous giant cells (arrowhead) and a thin epidermis (blue arrow); (C) Detail of the lesion observed in B, with giant cells (arrowhead) and macrophages (green arrows) containing amastigote forms of*Leishmania*spp.

## Discussion

Feline Leishmaniasis (FeL) has been previously reported in Teresina, Piauí (Batista et al., 2020); however, histopathological studies on organs of infected felines remain scarce. Research on dogs and other animals is often used as a reference for understanding the histopathological alterations (Grevot et al., 2005). Early diagnosis in felines is challenging due to the nonspecificity of clinical signs, which are frequently confused with those of other diseases.

Although felines exhibit comparative resistance to dogs, resulting in a lower prevalence of infection and clinical manifestations of*Leishmania*(Fernandez-Gallego et al., 2020), cases that progress to the clinical phase generally present a characteristic spectrum of signs. According to the literature, this clinical picture in felines includes cutaneous, ocular, and oral lesions, lymphadenopathy, weight loss, anorexia, and lethargy (Rocha et al., 2023). Dermatological alterations stand out as the most frequent, being present in approximately 75% of clinical cases (Fernandez-Gallego et al., 2020; Nascimento et al., 2024). The clinical presentation may combine cutaneous and systemic signs (Mendonça et al., 2017; Arenales et al., 2018), with skin lesions often associated with lymphadenopathy and nonspecific signs such as lethargy, anorexia, and weight loss (Brianti et al., 2019).

In the present study, the findings align with this profile, with the main manifestations being lymphadenomegaly, skin lesions, weight loss, and the presence of nodules, which corroborates reports from different regions that also highlight the high frequency of skin lesions in felines (Vides et al., 2011; Abramo et al., 2021).

Cutaneous lesions in felines are characterized by a spectrum that includes nodules, alopecia, scaling, and ulcers (Di Mattia et al., 2018), differing from canine leishmaniasis, where exfoliative dermatitis is more common, followed by ulcerative and nodular dermatitis (Saridomichelakis & Koutinas, 2014). All felines in this study exhibited at least one cutaneous clinical sign, similar to findings in canines, where ulcerative lesions are frequent (Silva et al., 2016). These findings suggest that, despite the similarities between the two forms of the disease, dermatological manifestations may differ between the species.

Despite the increase in FeL cases, few studies have evaluated the cellular immune response in these animals (Priolo et al., 2019). Studies demonstrate that felines develop a unique and predominantly Th1-type immune response against*Leishmania*spp., distinct from that observed in canine Leishmaniasis. This response is characterized by the production of IL-12 and IFN-γ, which act together to eliminate the parasite within cells, being crucial for controlling parasitic replication (Lode et al., 2025). However, this defense mechanism may not be entirely effective, as the parasite causing FeL has been reported to develop resistance to both the host's immune system and available treatments (Iatta et al., 2019).

The well-known dermotropism of the parasite in dogs, considered the main domestic reservoirs, results in a high parasitic load in the skin, making the parasite available to the vector (Ribeiro et al., 2024; Scorza et al., 2021), which manifests primarily in the skin, where the interaction between the parasite and the immune system occurs (Saridomichelakis, 2009). This study confirms that felines, although with distinct clinical manifestations, can present a significant cutaneous parasitic load, indicating their potential as reservoirs and a source of infection for sandflies. However, the presence of parasites in the skin is not the only determining factor for transmission, as asymptomatic animals, both dogs and felines, also demonstrate the capacity to infect vectors (Laurenti et al., 2013).

The detection of*Leishmania*spp. amastigotes by histopathology and parasitology in this study directly confirms the occurrence of a significant cutaneous parasitic load in felines. This finding corroborates the premise that felines have the potential to act as a source of infection for sandflies, aligning with the established principle in dogs that the parasitic density in the skin is a crucial parameter for transmission risk (Pereira-Fonseca et al., 2017).

The inflammatory infiltrate observed in the histopathology resembles that described in symptomatic dogs, where a high parasitic load is associated with a more intense inflammatory response (Verçosa et al., 2012; De Queiroz et al., 2011). The detection of amastigotes in feline skin corroborates the premise that this tissue constitutes a site of choice not only for diagnosis but also as a potential source of parasites for sandflies during blood meals (Ribeiro et al., 2024). Thus, the study provides direct anatomo-pathological evidence that supports the potential role of felines in the epidemiological chain of leishmaniasis.

The findings of the present study, documented through images, validate that the same fundamental immunopathological process described in dogs and humans is replicated in felines. The visualization of the inflammatory infiltrate in the skin of the studied felines confirms that infection by*Leishmania*spp. also triggers, in this species, an initial local immune response characterized by the recruitment of cells to the inoculation site, as proposed by Maran et al. (2016). This morphological evidence corroborates the premise that the skin is a key organ in the pathogenesis of feline leishmaniasis, and that the parasite-host interaction at this site is decisive for directing the immune response and, consequently, for the spectrum of clinical manifestations that may develop, from the asymptomatic to the polysymptomatic state (Baneth et al., 2008; Michel et al., 2011).

In dogs, the skin of the ear is a notable site for presenting a high parasitic load and a more intense and diffuse inflammatory process. This characteristic is favored by a greater density and diameter of blood vessels in the region, which facilitates the dissemination of the parasite (Ribeiro et al., 2024; Jacintho et al., 2018). In the present study, the cytological confirmation of*Leishmania*spp. amastigote forms in the ear of felines corroborates this susceptibility, aligning with reports pointing to the head, ear, and muzzle as the most commonly affected areas. This predisposition is often attributed to the lower hair density and greater exposure to vector sandflies (Silveira-Neto et al., 2015).

The high susceptibility of the ear, also extensively documented in dogs, may be related to additional factors, such as self-trauma, which stimulates itching and subsequently the accumulation of inflammatory cells (Tafuri et al., 2004), as well as its exposed anatomy. Detailed anatomical studies reveal greater parasitism at the tip of the ear compared to its lower third (Moura et al., 2008). It is believed that the higher incidence at the ear tip is attributed to its more exposed anatomy and a less effective cutaneous barrier, which facilitate infestation by the parasites, a finding confirmed by the present study.

In the histopathological pattern of the skin of infected felines, mononuclear cells predominated in fragments from the ear, back, and muzzle, corroborating findings in canines, where macrophages, lymphocytes, and plasma cells are frequent (Verçosa et al., 2012). The presence of granulomas, both in dogs and suggested in the felines, indicates an immune response directed at eliminating the parasites, characterized by epithelioid cells, lymphocytes, plasma cells, and granulocytes (Tafuri et al., 1996). The epidermis of the studied felines exhibited exocytosis, acanthosis, spongiosis, parakeratosis, and ulcers, which is partially consistent with findings from studies in canines, which describe acanthosis, hyperkeratosis, and ulcerations (Jacintho et al., 2018).

In summary, the dermatological and histopathological findings in FeL present important parallels with canine leishmaniasis, particularly regarding the cutaneous tropism of the parasite and the local inflammatory response. The skin, especially the ear region, confirms itself as a crucial site for the diagnosis, understanding of pathogenesis, and assessment of the disease transmission potential in felines, warranting greater attention in future studies.

## Conclusion

This study concluded that felines can become infected and exhibit inflammatory alterations and infiltrates similar to those observed in infected canines. The ear skin was identified as the most frequently parasitized tissue, highlighting its importance as a preferred site for diagnosis. The presence of a large number of amastigotes on the animal skin reinforces the potential role of felines in disease epidemiology. However, the intensity of inflammatory infiltrates and the presence of amastigotes were not directly related to the number of clinical signs exhibited by the animals. This study may aid in the early diagnosis, proper management, and prevention of FeL in endemic areas and provide relevant information on its zoonotic potential.

## Data Availability

All data necessary to support the results and conclusions of this study have been included in full within the body of the article.
